# Cytokine profiles in the joint depend on pathology, but are different between synovial fluid, cartilage tissue and cultured chondrocytes

**DOI:** 10.1186/s13075-014-0441-0

**Published:** 2014-09-26

**Authors:** Anika I Tsuchida, Michiel Beekhuizen, Marieke C ‘t Hart, Timothy RDJ Radstake, Wouter JA Dhert, Daniel BF Saris, Gerjo JVM van Osch, Laura B Creemers

**Affiliations:** Department of Orthopaedics, University Medical Center Utrecht, PO Box 85500, 3508 GA Utrecht, the Netherlands; Department of Rheumatology, Clinical Immunology & Laboratory of Translational Immunology, University Medical Center Utrecht, PO Box 85500, 3508 GA Utrecht, the Netherlands; Faculty of Veterinary Medicine, University of Utrecht, Yalelaan 1, 3584 CL Utrecht, the Netherlands; MIRA institute, Department of Tissue Regeneration, University of Twente, PO Box 217, 7500 AE Enschede, the Netherlands; Department of Orthopaedics and department of Otorhinolaryngology, Erasmus MC, University Medical Center, Po Box 2040, 3000 CA Rotterdam, the Netherlands; Department of Otorhinolaryngology, Erasmus MC, University Medical Center, Rotterdam, the Netherlands

## Abstract

**Introduction:**

This study aimed to evaluate whether profiles of several soluble mediators in synovial fluid and cartilage tissue are pathology-dependent and how their production is related to *in vitro* tissue formation by chondrocytes from diseased and healthy tissue.

**Methods:**

Samples were obtained from donors without joint pathology (*n* = 39), with focal defects (*n* = 65) and osteoarthritis (*n* = 61). A multiplex bead assay (Luminex) was performed measuring up to 21 cytokines: Interleukin (IL)-1α, IL-1β, IL-1RA, IL-4, IL-6, IL-6Rα, IL-7, IL-8, IL-10, IL-13, tumor necrosis factor (TNF)α, Interferon (IFN)γ, oncostatin M (OSM), leukemia inhibitory factor (LIF), adiponectin, leptin, monocyte chemotactic factor (MCP)1, RANTES, basic fibroblast growth factor (bFGF), hepatocyte growth factor (HGF), vascular growth factor (VEGF).

**Results:**

In synovial fluid of patients with cartilage pathology, IL-6, IL-13, IFNγ and OSM levels were higher than in donors without joint pathology (*P* ≤0.001). IL-13, IFNγ and OSM were also different between donors with cartilage defects and OA (*P* <0.05). In cartilage tissue from debrided defects, VEGF was higher than in non-pathological or osteoarthritic joints (*P* ≤0.001). IL-1α, IL-6, TNFα and OSM concentrations (in ng/ml) were markedly higher in cartilage tissue than in synovial fluid (*P* <0.01). Culture of chondrocytes generally led to a massive induction of most cytokines (*P* <0.001). Although the release of inflammatory cytokines was also here dependent on the pathological condition (*P* <0.001) the actual profiles were different from tissue or synovial fluid and between non-expanded and expanded chondrocytes. Cartilage formation was lower by healthy unexpanded chondrocytes than by osteoarthritic or defect chondrocytes.

**Conclusions:**

Several pro-inflammatory, pro-angiogenic and pro-repair cytokines were elevated in joints with symptomatic cartilage defects and/or osteoarthritis, although different cytokines were elevated in synovial fluid compared to tissue or cells. Hence a clear molecular profile was evident dependent on disease status of the joint, which however changed in composition depending on the biological sample analysed. These alterations did not affect *in vitro* tissue formation with these chondrocytes, as this was at least as effective or even better compared to healthy chondrocytes.

## Introduction

Soluble mediators, such as cytokines, chemokines, adipokines and growth factors, are key regulators of cartilage metabolism. Under homeostatic conditions the production of these mediators is tightly regulated. In many joint diseases, including osteoarthritis (OA) and joint trauma, elevated levels of inflammatory cytokines are present [1] but their pathophysiological role is not always well established. In inflammatory diseases such as rheumatoid arthritis, the synovial cells produce large quantities of inflammatory cytokines and degradative enzymes [2], which reach the cartilage through the synovial fluid. Although less outspoken, synovial inflammation is also present in OA [3,4] and sometimes in joints with cartilage defects [5], and the balance between anabolic and catabolic mediators has been suggested to be in favour of catabolic factors leading to a net breakdown of cartilage. In addition to their possible role in the pathophysiology of joint disease, soluble mediators present in the joint may also affect cartilage repair, both in OA and in cartilage defect treatment [6,7].

These mediators are not only produced by the synovial lining cells, but also by resident chondrocytes in response to both biological and mechanical stimuli [1]. In fact, in OA certain cytokines appeared to be predominantly produced by cartilage rather than synovial tissue [8]. Furthermore, some mediators were shown to bind to extracellular matrix components, such as glycosaminoglycans (GAGs), in cartilage [9]. Local concentrations in the microenvironment of the chondrocyte might therefore differ from those in the synovial fluid. Evaluating the presence of mediators in the cartilage tissue in addition to the synovial fluid might provide more insight into the possible mechanisms of cartilage regeneration and degeneration.

In addition to the cytokines in synovial fluid and cartilage extracellular matrix, studying the cytokine profile during *in vitro* cartilage formation may provide essential clues as to which mediators are important for tissue engineering of cartilage and to what extent cytokine production by chondrocytes may affect the outcome of cartilage repair procedures. Few studies have directly compared healthy chondrocytes with chondrocytes obtained from cartilage defects and/or osteoarthritic chondrocytes in terms of regenerative capacity [10] and a comprehensive secretory profile is lacking. Characterising the mediators produced by both healthy and diseased chondrocytes may allow for more specific inhibition of mediators in order to improve cartilage regeneration, especially by dedifferentiated or diseased chondrocytes.

In this study we therefore compared the presence of soluble mediators commonly suggested to play a role in joint pathology in synovial fluid and cartilage tissue for donors with healthy cartilage, patients with symptomatic cartilage defects and patients with end-stage OA. In addition, the production of these and additional cytokines by isolated chondrocytes during *in vitro* cartilage formation was investigated using both expanded and nonexpanded chondrocytes obtained from donors with different joint pathology. Clustering was investigated by principal component analysis to verify whether the roles and pathways for these cytokines are likely to be similar in the various all of these biological samples assayed.

## Methods

### Synovial fluid and cartilage sample collection and cell isolation

Collection of all patient material and all aspects of the study protocol were performed according to the Medical Ethical regulations of the University Medical Centre Utrecht and according to the ‘good use of redundant tissue for clinical research’ guideline constructed by the Dutch Federation of Medical Research Societies on collection of redundant tissue for research [11]. This study does not meet the definition of human subject research or require informed consent. Anonymous use of redundant tissue for research purposes is part of the standard treatment agreement with patients in our hospital [12].

Macroscopically healthy articular cartilage and synovial fluid was obtained from donors within 24 hours post mortem (see Table 1 for details). Defect cartilage and synovial fluid was obtained from donors undergoing either microfracture or autologous chondrocyte implantation for focal grade III and grade IV cartilage defects. During those procedures, the cartilage defect is debrided to remove all cartilage remnants down to the subchondral bone and create a stable cartilage rim. The debrided cartilage was used. Of the 31 patients with symptomatic cartilage defects, one had associated anterior cruciate ligament injury and a history of partial menisectomy, another three had received previous partial menisectomies and one an anterior cruciate ligament reconstruction. OA cartilage and synovial fluid was obtained from donors undergoing total knee arthroplasty. Synovial fluid was spun down at 300 × *g* to remove debris, and stored at –80°C until use or analysis.

**Table 1:** **Donor characteristics**

**Donor type**	**Synovial fluid**	**Tissue**	**Nonexpanded chondrocytes**	**Expanded chondrocytes**
Healthy	*n* = 20	*n* = 9^a^	*n* = 8^a^	*n* = 15
Age, average (range)	40 (25 to 47)	69 (54 to 81)	66 (54 to 75)	67 (54 to 81)
% male	Unknown	80	50	60
Cartilage defect	*n* = 31	*n* = 16	*n* = 10	*n* = 13
Age, average (range)	33 (20 to 48)	33 (16 to 45)	32 (18 to 44)	24 (19 to 36)
% male	Unknown	47	70	85
Osteoarthritis	*n* = 27	*n* = 27	*n* = 12	*n* = 13
Age, average (range)	70 (53 to 81)	67 (46 to 90)	69 (49 to 83)	70 (46 to 83)
% male	Unknown	23	38	38

^a^Of four healthy donors, both tissue and nonexpanded chondrocytes were analysed; for all the other donors, either synovial fluid, tissue, nonexpanded or expanded cells were analysed.

Cartilage samples were rinsed in phosphate-buffered saline (PBS), and either snap frozen in liquid nitrogen and stored at –80°C until later protein extraction or cut into small pieces and enzymatically digested overnight at 37°C in 0.15% collagenase type II (Worthington, Lakewood, NJ, USA) in Dulbecco’s modified Eagle’s medium (Gibco, Life Technologies, Bleiswijk, the Netherlands) with penicillin/streptomycin (100 U/ml/100 μg/ml; Invitrogen, Life Technologies). After digestion, the cell suspension was filtered through a 70 μm cell strainer (BD Biosciences, San Diego CA, USA), and the chondrocytes were spun down by 10 minutes of centrifugation at 300 × *g*.

### Protein extraction

The frozen cartilage samples (Table 1) were cut into 7 μm sections with a cryomicrotome. The sections were collected and carefully weighed. Approximately 100 mg cartilage tissue was collected per sample and lysed in 5 μl lysis buffer per milligram of tissue with complete protease inhibitor (Complete Lysis M, #04719964001; Roche Diagnostics, Almere, the Netherlands). The samples were cooled on ice and homogenised using 1.4 mm ceramic beads (VWR, Amsterdam, the Netherlands) in a bead beater. Subsequently, the samples were centrifuged at 4°C for 4 minutes at 20,217 × *g* and supernatants collected. Supernatants were then filtered by centrifuging through a polypropylene tube containing a 0.22 μm nylon membrane (Spin-X column; Corning, Amsterdam, the Netherlands) prior to measurements of the cytokine levels.

### Measurement of cytokine levels

To determine the cytokine levels in the synovial fluids and tissue extracts of healthy, defect and OA donors and in the conditioned media (day 7) of healthy, defect and OA chondrocytes during *in vitro* tissue formation, a multiplex bead-assay (Luminex, Luminex Corporation, Austin TX, USA) was performed as described previously [13,14]. A panel of a total 21 cytokines was available – interleukin (IL)-1α, IL-1β, IL-1RA, IL-4, IL-6, IL-6Rα, IL-7, IL-8, IL-10, IL-13, tumour necrosis factor alpha (TNFα), interferon gamma (IFNγ), oncostatin M (OSM), leukaemia inhibitory factor (LIF), adiponectin, leptin, monocyte chemotactic factor 1 (MCP1), RANTES, basic fibroblast growth factor (bFGF), hepatocyte growth factor (HGF), and vascular endothelial growth factor (VEGF) – of which 11 were measured in the synovial fluid, 19 in tissue extracts and all 21 in conditioned media. Briefly, specific antibodies were coupled to carboxylated beads (Luminex Corporation, Austin, TX, USA). The levels of IL-6 present in the synovial fluid have been published previously [15]. Recombinant cytokines were used to make a standard curve. Synovial fluid was first treated with hyaluronidase (type IV-S; Sigma-Aldrich, Zwijndrecht, the Netherlands) at a concentration of 20 U/ml for 30 minutes at 37°C and then filtered by centrifuging through a Spin-X column. Subsequently, the synovial fluid samples were diluted 1:2 with HPE-0.1375% Tween (Sanquin, Amsterdam, the Netherlands). To block possible interfering antibodies present in the synovial fluid, the samples were diluted with an equal volume of 10% (v/v) normal rat and mouse serum (Rockland Immunochemicals Inc., Gilbertsville, PA, USA). Tissue extracts and medium samples were directly incubated with the coupled beads. After incubation with the appropriate biotinylated antibodies, samples were thoroughly washed and incubated with streptavidin–phycoerythrin (BD Biosciences) for 10 minutes. After washing, the samples were measured and analysed using the Bio-Plex suspension system (Bio-Rad Laboratories, Hercules, CA, USA) with Bio-Plex Manager software, version 3.0. The concentration of cytokines was expressed as picograms per microlitre using the standard curves. Results of specific enzyme-linked immunosorbent assays for determination of cytokine levels have previously been shown to be comparable with the multiplex bead assay (Luminex) [8].

### Chondrocyte-mediated tissue production *in vitro*

Isolated chondrocytes from healthy, defect and OA cartilage were used either directly after isolation or after expansion to passage 2 (see Table 1 for more details). Expansion was performed in monolayers at 37°C and 5% carbon dioxide at a seeding density of 5,000 cells/cm^2^ in expansion medium consisting of Dulbecco’s modified Eagle’s medium, 10% foetal bovine serum (Hyclone, Thermo Scientific, Etten-Leur, the Netherlands), penicillin/streptomycin (100 U/ml/100 μg/ml) and 10 ng/ml bFGF (R&D Systems, Minneapolis, MN, USA). Unexpanded chondrocytes and expanded chondrocytes were seeded on collagen type II-coated (Chicken sternal cartilage, #C9301; Sigma-Aldrich) Millicell filters (Millipore Co., Bedford, MA, USA), at 1.6 × 10^6^ cells/cm^2^ and redifferentiated during 28 days in redifferentiation medium consisting of Dulbecco’s modified Eagle’s medium, 0.2 mM L-ascorbic acid-2-phosphate (AsAp; Sigma-Aldrich), 2% human serum albumin (Sanquin), penicillin/streptomycin (100 U/ml/100 μg/ml), 2% insulin–transferrin–selenium-X (Invitrogen) and 5 ng/ml transforming growth factor beta 2 (R&D systems).

### Glycosaminoglycan and DNA analysis

After culture, the formed tissue was digested overnight in a papain buffer (250 μg/ml papain (Sigma-Aldrich) in 50 mM ethylenediamine tetraacetic acid and 5 mM L-cysteine) at 56°C, followed by determination of the GAG content using the dimethylmethylene blue assay [16]. The ratio of absorption at 540 nm to that at 595 nm was used to calculate the GAG content, using chondroitin sulphate (shark; Sigma-Aldrich) as a standard. Supernatants were also collected and analysed for GAGs released into the medium.

The DNA content was determined from the papain digest using a Picogreen DNA assay (Invitrogen) in accordance with the manufacturer’s instructions.

### Histological evaluation

Newly formed cartilage on filters was fixed in 4% buffered formaldehyde, dehydrated in alcohol, rinsed in xylene and infiltrated and embedded with paraffin. For histology, 5 μm sections were deparaffinised in xylene, rehydrated in alcohol and stained with safranin-O (Merck, Darmstadt, Germany) for GAG and counterstained with Weigert’s haematoxylin (Klinipath, Duiven, the Netherlands) and 0.4% fast green (Merck) for nuclei and cytoplasm, respectively.

The deposition of type II collagen was evaluated by immunohistochemistry. Antigen retrieval was performed by 1 mg/ml pronase (Sigma-Aldrich) for 30 minutes at 37°C followed by hyaluronidase incubation for 30 minutes at 37°C. After antigen retrieval, the sections were blocked using a PBS–bovine serum albumin 5% solution for 30 minutes followed by overnight incubation at 4°C with primary antibody against human type II collagen (II-II6B3 1/100 in 5% PBS/bovine serum albumin; Hybridomabank, Ioway City, Iowa, USA). A biotinylated secondary anti-mouse antibody was used (1/200 in 5% PBS/bovine serum albumin; GE Healthcare, Pollards Wood, UK) for 1 hour at room temperature, followed by incubation with streptavidin/peroxidase (1/500 in 5% PBS/bovine serum albumin; Beckman Coulter, Brea, CA, USA) for 1 hour at room temperature. Antibody binding was visualised using 3-diaminobenzidine (Sigma-Aldrich). All immunohistochemical sections were counterstained using Mayer’s haematoxylin.

### Statistical analysis

All statistical analyses were performed using SPSS 18.0 (SPSS Inc., Chicago, IL, USA). Results are displayed as mean ± standard deviation. Cytokine levels were not distributed normally, therefore differences in cytokine levels between healthy, cartilage defect and OA donors were determined using the Kruskall–Wallis test with *post hoc* Mann–Whitney *U* test. Differences in cytokine levels between synovial fluid and tissue were determined using the Mann–Whitney *U* test, and those between tissue, unexpanded and expanded cells with the Kruskall–Wallis test. Principal component analysis was performed to reduce correlated cytokines to a smaller set of uncorrelated factors delineating subsets of cytokines. Only cytokines with communalities >0.5 were included. Loading factors were maximised using Varimax rotation with Kaiser normalisation. The number of clusters was decided based on the scree plot and eigenvalues (>1.0). Cytokines were categorised per cluster when their loading scores were >0.5. A test for Cronbach’s alpha was performed on each cluster to verify consistency. Separate principal component analysis of healthy, cartilage defect and OA samples was not possible due to the limited number of subjects per disease condition. Differences in GAG and DNA content between healthy, cartilage defect and OA donors were determined using analysis of variance with *post hoc t* test, and differences between unexpanded and expanded chondrocytes by *t* test. Multiple linear regression analysis with backward elimination was performed to identify cytokines that best predict cartilage regeneration as measured by GAG/DNA.

## Results

### Elevated levels of IL-6, IL-13, IFNγ, OSM and VEGF in donors with symptomatic focal cartilage defects

In total, 11 soluble mediators were measured in the synovial fluid of healthy donors, donors with symptomatic cartilage defects and osteoarthritic donors. Four of them were present at significantly different concentrations, namely IL-6, IL-13, IFNγ and OSM (*P* ≤0.001; Table 2). The concentrations of IL-6 and OSM were significantly lower in synovial fluid of donors without known joint pathology than in synovial fluid of patients with cartilage defects or OA (in line with previous data [15]). The concentrations of IL-13 and IFNγ were highest in synovial fluid of patients with a cartilage defect.

**Table 2:** **Cytokines in the synovial fluid and cartilage tissue**

**Synovial fluid**					**Cartilage**				
**Cytokines (per ml SF)**	**Healthy (** ***n*** **= 20)**	**Defect (** ***n*** **=31)**	**Osteoarthritis (** ***n*** **= 27)**	***P*** **value**	**Cytokines per g tissue**	**Healthy (** ***n*** **= 9)**	**Defect (** ***n*** **= 16)**	**Osteoarthritis (** ***n*** **= 27)**	***P*** **value**
IL-1α (pg/ml)	16 ± 10 (100%)	14 ± 8 (100%)	15 ± 22 (89%)	0.085	IL-1α (pg/g)	5 ± 13 (22%)	366 ± 706 (25%)	183 ± 391 (26%)	0.470
IL-1β (pg/ml)	1 ± 2 (25%)	15 ± 18 (55%)	8 ± 16 (41%)	0.032	IL-1β (pg/g)	0 ± 0 (0%)	23 ± 63 (13%)	0 ± 1 (4%)	0.336
IL-4 (pg/ml)	0 ± 0 (0%)	2 ± 3 (32%)	1 ± 4 (15%)	0.026	IL-4 (pg/g)	0 ± 0 (0%)	1 ± 2 (13%)	0 ± 1 (7%)	0.261
IL-6^a^ (pg/ml)	64 ± 120 (40%)	261 ± 385 (96%)	396 ± 508 (82%)	<0.001*	IL-6 (pg/g)	1064 ± 3193 (11%)	1473 ± 3162 (19%)	935 ± 2739 (19%)	0.752
IL-7 (pg/ml)	0 ± 0 (0%)	0 ± 0 (0%)	5 ± 28 (4%)	0.459					
IL-8 (pg/ml)	25 ± 29 (75%)	27 ± 33 (59%)	52 ± 95 (85%)	0.352	IL-8, ng/g	12 ± 20 (67%)	10 ± 30 (31%)	15 ± 44 (48%)	0.442
IL-10 (pg/ml)	1 ± 6 (5%)	0 ± 0 (0%)	9 ± 35 (15%)	0.134	IL-10 (pg/g)	0 ± 0 (0%)	812 ± 2113 (13%)	578 ± 1650 (15%)	0.400
IL-13 (pg/ml)	1 ± 2 (5%)	38 ± 41 (64%)	18 ± 40 (33%)	<0.001*	IL-13 (pg/g)	0 ± 0 (0%)	799 ± 1641 (25%)	250 ± 648 (15%)	0.121
TNFα (pg/ml)	0 ± 0 (0%)	2 ± 8 (9%)	4 ± 20 (11%)	0.324	TNFα (pg/g)	522 ± 1084 (22%)	3069 ± 4453 (25%)	1733 ± 3212 (30%)	0.467
IFNγ (pg/ml)	47 ± 17 (100%)	68 ± 38 (100%)	51 ± 69 (93%)	0.001*	IFNγ (pg/g)	0 ± 0 (0%)	226 ± 848 (13%)	0 ± 0 (0%)	0.101
OSM (pg/ml)	2 ± 7 (5%)	22 ± 46 (64%)	38 ± 121 (22%)	0.001*	OSM (pg/g)	66 ± 108 (44%)	170 ± 241 (38%)	220 ± 424 (37%)	0.786
					LIF (pg/g)	0 ± 0 (0%)	2797 ± 6515 (13%)	3529 ± 10164 (22%)	0.279
					Adiponectin (μg/g)	12 ± 10 (100%)	57 ± 62 (88%)	19 ± 29 (100%)	0.002
					Leptin (ng/g)	15 ± 29 (33%)	120 ± 169 (63%)	112 ± 136 (83%)	0.026
					MCP1 (pg/g)	182 ± 423 (22%)	1336 ± 1711 (50%)	1249 ± 1250 (68%)	0.044
					RANTES (pg/g)	0 ± 0 (0%)	1225 ± 2163 (31%)	510 ± 1121 (22%)	0.056
					bFGF (ng/g)	129 ± 335 (44%)	439 ± 691 (69%)	615 ± 719 (74%)	0.055
					HGF (pg/g)	3171 ± 6757 (56%)	5824 ± 5110 (69%)	2986 ± 3452 (74%)	0.113
					VEGF (ng/g)	19 ± 11 (100%)	212 ± 149 (88%)	49 ± 28 (100%)	<0.001*
					IL-1RA (pg/g)	495 ± 1017 (22%)	1856 ± 3172 (25%)	2504 ± 10792 (33%)	0.573

The presence of cytokines was measured in synovial fluid (SF) and cartilage tissue extracts from healthy donors and donors with symptomatic cartilage defects and osteoarthritic donors. Results are represented as mean ± standard deviation (% of samples in which the cytokine was present). Differences in cytokine levels between healthy, cartilage defect and OA donors were determined using the Kruskall–Wallis test (*P* values shown in table) with *post hoc* Mann–Whitney *U* test: synovial fluid – IL-6: H vs. CD, *P* <0.001; H vs. OA, *P* = 0.001; IL-13: H vs. CD, *P* <0.001; H vs. OA, *P* = 0.018; CD vs. OA, *P* = 0.029; IFNγ: H vs. CD, *P* = 0.008; H vs. OA, *P* = 0.05; CD vs. OA, *P* = 0.001; OSM: H vs. CD, *P* <0.001; CD vs. OA, *P* = 0.030; tissue – VEGF: H vs. CD, *P* <0.001; H vs. OA, *P* = 0.003; CD vs. OA, *P* <0.001. bFGF, basic fibroblast growth factor; CD, cartilage defect; H, healthy; HGF, hepatocyte growth factor; IFNγ, interferon gamma; IL, interleukin; LIF, leukaemia inhibitory factor; MCP1, monocyte chemotactic factor 1; OA, osteoarthritis; OSM, oncostatin M; RANTES, regulated upon activation normal T cell expressed and presumably secreted; TNFα, tumour necrosis factor alpha; VEGF, vascular endothelial growth factor. *P <0.001. ^a^Data published previously [15].

A total of 19 soluble mediators were measured in cartilage tissue extracts of healthy cartilage, cartilage debrided from defects and osteoarthritic cartilage (Table 3). Only VEGF concentrations were statistically significantly different, with the highest concentrations in cartilage debrided from defects and the lowest concentrations in healthy cartilage and a significant difference between OA and defect cartilage (*P* <0.001; Tables 2 and 3).

**Table 3:** **Cytokines produced by chondrocytes in native tissue and in culture per cell**

	**Tissue**				**Unexpanded chondrocytes**				**Expanded chondrocytes**			
**Cytokine (per mg DNA)**	**Healthy (** ***n*** **= 9)**	**Defect (** ***n*** **= 16)**	**Osteoarthritis (** ***n*** **= 27)**	***P*** **value**	**Healthy (** ***n*** **= 8)**	**Defect (** ***n*** **= 10)**	**Osteoarthritis (** ***n*** **= 12)**	***P*** **value**	**Healthy (** ***n*** **= 15)**	**Defect (** ***n*** **= 13)**	**Osteoarthritis (** ***n*** **= 13)**	***P*** **value**
IL-1α (pg/mg)	5 ± 12	198 ± 630	408 ± 1,062	0.844	439 ± 268	993 ± 743	460 ± 271	0.006	1,718 ± 2,689	1,978 ± 1,464	2,569 ± 2,528	0.006
IL-1β (pg/mg)	0 ± 0	14 ± 41	0 ± 2	0.336	14 ± 33	816 ± 968	125 ± 151	<0.001*	283 ± 368	369 ± 270	459 ± 408	0.010
IL-4 (pg/mg)	0 ± 0	0 ± 1	0 ± 1	0.536	18 ± 49	18 ± 40	40 ± 76	0.691	148 ± 224	200 ± 163	263 ± 259	0.002
IL-6 (pg/mg)	4,813 ± 14,438	678 ± 1,867	1,840 ± 6,389	0.928	1,715 ± 397	1,739 ± 437	1,918 ± 77	0.037	423 ± 414	136 ± 268	721 ± 319	<0.001*
IL-7 (pg/mg)					1,288 ± 378	1,120 ± 547	1,304 ± 619	0.457	300 ± 401	411 ± 343	545 ± 555	0.008
IL-8 (ng/mg)	39 ± 94	7 ± 23	28 ± 102	0.192	1,070 ± 572	786 ± 625	784 ± 399	0.036	28 ± 36	36 ± 43	113 ± 85	<0.00*
IL-10 (pg/mg)	0 ± 0	252 ± 783	969 ± 3,344	0.483	3,021 ± 2,455	16,529 ± 20,669	4,925 ± 3,429	0.015	5,200 ± 6,788	6,524 ± 4,806	9,471 ± 9,333	0.029
IL-13 (ng/mg)	0 ± 0	378 ± 783	545 ± 1,531	0.296	2,027 ± 597	3,249 ± 1,155	2,936 ± 1,220	0.002	4,486 ± 6,088	5,614 ± 3,730	6,787 ± 5,935	0.006
TNFα (pg/mg)	499 ± 1,020	2,054 ± 4,520	3,567 ± 8,065	0.714	253 ± 184	414 ± 346	426 ± 452	0.319	183 ± 221	217 ± 148	310 ± 315	0.154
IFNγ (pg/mg)	0 ± 0	175 ± 677	0 ± 0	0.101	50 ± 214	54 ± 245	173 ± 636	0.615	538 ± 866	720 ± 767	1,036 ± 1,287	0.079
OSM (pg/mg)	156 ± 279	135 ± 239	394 ± 776	0.963	111 ± 164	2,736 ± 3,990	369 ± 640	0.138	26 ± 37	26 ± 23	46 ± 55	0.135
LIF (ng/mg)	0 ± 0	1 ± 4	5 ± 13	0.238	76 ± 50	194 ± 127	97 ± 56	0.005	38 ± 37	23 ± 16	48 ± 24	0.000*
Adiponectin (ng/mg)	15,700 ± 12,550	60,900 ± 74,720	20,000 ± 26,960	0.238	1,582 ± 903	3,689 ± 5,136	1,777 ± 1,220	0.723	20 ± 31	22 ± 21	36 ± 41	0.022
Leptin (ng/mg)	14 ± 29	75 ± 151	161 ± 199	0.009	22 ± 7	26 ± 7	22 ± 9	0.125	22 ± 24	29 ± 18	35 ± 27	0.014
MCP1 (pg/mg)	672 ± 1,890	806 ± 1,067	2,216 ± 3,780	0.068	376 ± 148	626 ± 474	331 ± 219	0.065	43 ± 14	38 ± 20	53 ± 49	0.176
RANTES (pg/mg)	0 ± 0	986 ± 2,687	1,074 ± 2,852	0.208	129 ± 149	63 ± 80	106 ± 89	0.057	3 ± 4	1 ± 1	4 ± 4	0.034
bFGF (ng/mg)	196 ± 527	514 ± 762	760 ± 923	0.089	12 ± 15	39 ± 68	11 ± 18	0.427	20 ± 17	23 ± 14	34 ± 26	0.021
HGF (pg/mg)	3,262 ± 6,163	5763 ± 10,551	3,911 ± 4,661	0.565	151 ± 243	431 ± 470	353 ± 534	0.118	4,243 ± 6,533	5,314 ± 4,061	7,242 ± 7,595	0.004
VEGF (ng/mg)	33 ± 22	173 ± 133	66 ± 53	<0.001*	638 ± 468	828 ± 522	742 ± 298	0.333	126 ± 23	133 ± 29	124 ± 25	0.610
IL-1RA (pg/mg)	475 ± 960	990 ± 1938	1,871 ± 7,209	0.868	2,611 ± 2,167	20,259 ± 2,3520	11,005 ± 17,284	0.002	349 ± 260	433 ± 260	579 ± 442	0.031
IL-6Rα (pg/mg)					8,217 ± 2,190	5,397 ± 5,195	4,666 ± 5,094	0.037	3,276 ± 10,387	593 ± 324	456 ± 333	0.064

To compare the production of cytokines by chondrocytes in their native tissue (tissue extracts) and during culture (conditioned media day 7, both nonexpanded and expanded chondrocytes), cytokine production was evaluated per DNA in samples from healthy cartilage, debrided cartilage from defects and osteoarthritic cartilage. Results are represented as mean ± standard deviation. Differences in cytokine levels between healthy, cartilage defect and OA donors were determined using the Kruskall–Wallis test (*P* values shown in table) with *post hoc* Mann–Whitney *U* test: tissue – VEGF: H vs. CD, *P* = 0.002; H vs. OA, *P* = 0.024; CD vs. OA, *P* = 0.002; unexpanded chondrocytes – IL-1β: H vs. CD, *P* <0.001; H vs. OA, *P* = 0.002; CD vs. OA, *P* = 0.013; expanded chondrocytes – IL-6: H vs. CD, *P* <0.001; H vs. OA, *P* <0.001; CD vs. OA, *P* <0.001; IL-8: H vs. OA, *P* <0.001; CD vs. OA, *P* <0.001; H vs. OA, *P* = 0.008; CD vs. OA, *P* <0.001. bFGF, basic fibroblast growth factor; CD, cartilage defect; H, healthy; HGF, hepatocyte growth factor; IFNγ, interferon gamma; IL, interleukin; LIF, leukaemia inhibitory factor; MCP1, monocyte chemotactic factor 1; OA, osteoarthritis; OSM, oncostatin M; RANTES, regulated upon activation normal T cell expressed and presumably secreted; TNFα, tumour necrosis factor alpha; VEGF, vascular endothelial growth factor. *P <0.001.

To be able to compare the levels in synovial fluid and cartilage tissue, the wet weight of tissue was taken to represent to its volume according to an average of 1.05 g/ml density for tissues devoid of inorganic constituents [17] such as bone (Table 2). This would underestimate values by 5% when expressed per volume unit. Concentrations of IL-1α, IL-1β, IL-6, TNFα, IFNγ and OSM were different between synovial fluid and cartilage tissue (*P* <0.01), regardless of whether the tissue was diseased or not. However, especially in 1 ml cartilage debrided from defects and OA cartilage, some tissue explants revealed concentrations of IL-1α, IL-6, TNFα and OSM that were at least 10-fold to 100-fold higher than in 1 ml synovial fluid (Table 2).

### Cytokine production by isolated and cultured chondrocytes during *in vitro* tissue formation

When comparing cytokine presence per milligram of DNA in native tissue with the levels produced during *in vitro* tissue formation, cytokine production by chondrocytes in cell culture was generally much higher than the levels found in their native matrix (*P* <0.001 for all cytokines, except OSM *P* = 0.043; Table 3). There were no differences in DNA content between healthy, cartilage defect and OA tissue (*P* = 0.1) (Table 3). Exceptions were TNFα, adiponectin, bFGF and HGF, which were lower (*P* <0.001 for all cytokines) in cultured cells, and leptin, which was similar. IL-1β, IL-4, IL-10, IL-13, IFNγ, LIF and RANTES were not present in healthy cartilage tissue, but were induced during *in vitro* tissue formation.

Soluble mediator production by isolated but unexpanded chondrocytes during *in vitro* cartilage formation was very similar between healthy, cartilage defect and OA chondrocytes (Table 3). Only IL-1β was produced at significantly higher amounts by chondrocytes from debrided cartilage from defects compared with healthy and OA chondrocytes (*P* <0.001; Table 3).

Expansion in monolayers for two passages had a major effect on cytokine secretion. The secretion of most cytokines was reduced at least by one-half after expansion, but the expression of IL4, IFNγ, HGF and, to a lesser degree, IL-1α and IL-1β was clearly increased by expansion of chondrocytes (Table 3). Although before expansion only IL-1β was differentially released between chondrocytes from different joint pathologies, culture-expanded chondrocytes from OA cartilage showed a higher production of IL-6, IL-8 and LIF during regeneration culture compared with chondrocytes from healthy cartilage and/or cartilage debrided from defects (*P* <0.001; Table 3).

### Cartilage matrix formation during culture

**Figure 1:**
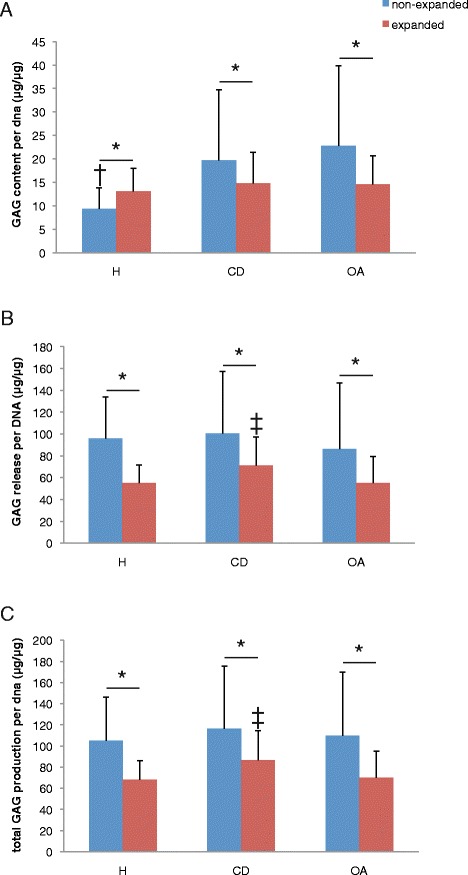
**Cartilage matrix production by healthy, cartilage defect and osteoarthritis chondrocytes.** Cartilage regeneration cultures of three healthy (H), three cartilage defect (CD) and three osteoarthritic (OA) donors, with either nonexpanded or expanded chondrocytes. **(A)** Glycosaminoglycan (GAG) content per DNA, **(B)** cumulative GAG release per DNA, and **(C)** total GAG production (GAG content + cumulative GAG release) per DNA after 28 days of culture. *P <0.05, †*P* <0.05 compared with CD and OA; ‡*P* <0.05 compared with H and OA.

**Figure 2:**
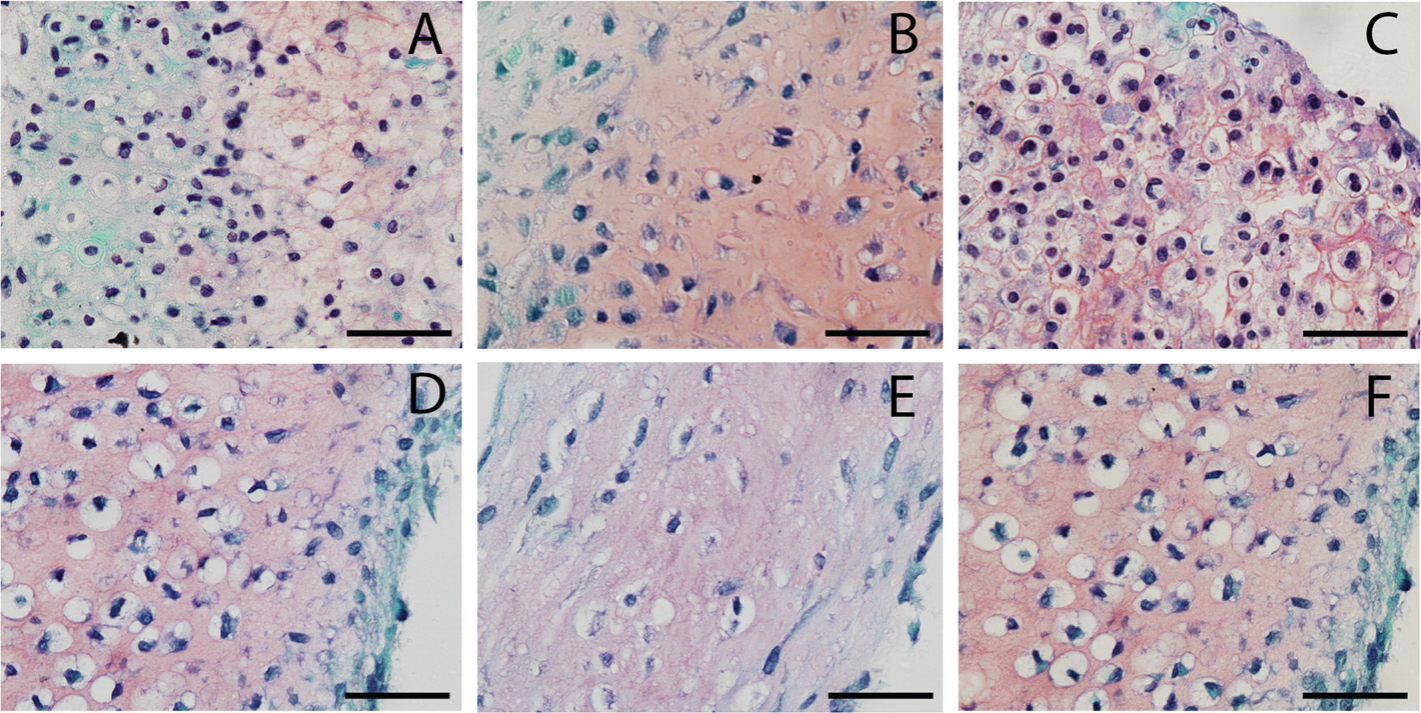
**Safranin O staining of newly formed tissue by healthy, cartilage defect and osteoarthritis chondrocytes.** Safranin O staining of newly formed tissue after redifferentiation of chondrocytes on type II collagen-coated filters after 28 days of culture. **(A)** Nonexpanded healthy chondrocytes, **(B)** nonexpanded cartilage defect chondrocytes, **(C)** nonexpanded osteoarthritic chondrocytes, **(D)** expanded healthy chondrocytes, **(E)** expanded cartilage defect chondrocytes, and **(F)** expanded osteoarthritic chondrocytes.

**Figure 3:**
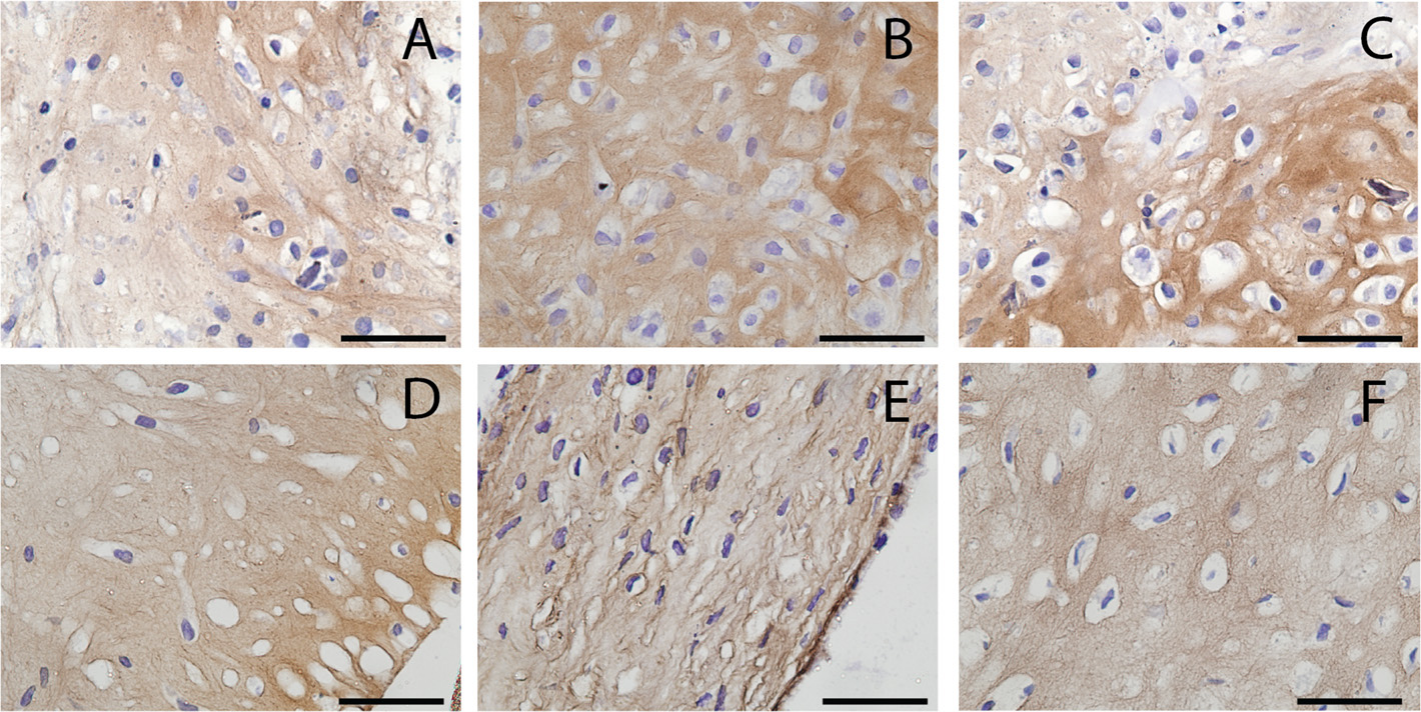
**Collagen type II in newly formed tissue by healthy, cartilage defect and osteoarthritis chondrocytes.** Collagen type II immunohistochemistry of newly formed tissue after redifferentiation of chondrocytes on type II collagen-coated filters after 28 days of culture. **(A)** Nonexpanded healthy chondrocytes, **(B)** nonexpanded cartilage defect chondrocytes, **(C)** nonexpanded osteoarthritic chondrocytes, **(D)** expanded healthy chondrocytes, **(E)** expanded cartilage defect chondrocytes, and **(F)** expanded osteoarthritic chondrocytes.

Total GAG production per DNA was not different between healthy, cartilage defect and OA nonexpanded chondrocytes (Figure 1C). However, most of the GAG produced during culture was not retained in the newly formed tissue but released in the medium (Figure 1B). Remarkably, unexpanded chondrocytes from defect cartilage (GAG/DNA, 19.6 ± 15.1 μg/μg) and OA chondrocytes (GAG/DNA, 22.8 ± 17.1 μg/μg) retained more of the produced GAG in the matrix than isolated unexpanded chondrocytes from healthy cartilage (GAG/DNA, 9.4 ± 4.4 μg/μg; *P* <0.001) (Figure 1A). However, we did not see this same difference in safranin-O staining intensity (Figure 2). Nonquantitative evaluation of collagen II deposition showed clear collagen II staining without apparent differences between donor types (Figure 3).

The GAG content per DNA of the newly formed cartilage by culture-expanded chondrocytes after 4 weeks of culture was lower than that by unexpanded chondrocytes and not different between chondrocytes isolated from healthy cartilage, cartilage defect or OA. In contrast, total GAG production per DNA was highest by expanded chondrocytes from cartilage debrided from defects (Figure 1C), which was associated with a higher GAG release by these cells (Figure 1B). The newly formed cartilage matrix by expanded cells contained collagen type II without apparent differences between donor types (Figure 3).

### Cluster analysis

**Figure 4:**
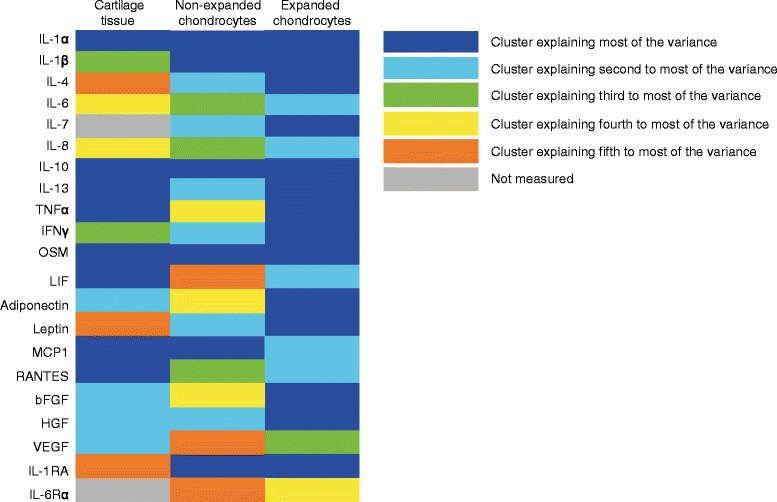
**Principle component analyses of cytokines in cartilage tissue and cells.** Principal component analysis of the measured cytokines in cartilage tissue and conditioned media (day 7) of nonexpanded and expanded chondrocytes during regeneration. Different clusters are indicated by different colours. bFGF, basic fibroblast growth factor; HGF, hepatocyte growth factor; IFNγ, interferon gamma; IL, interleukin; LIF, leukaemia inhibitory factor; MCP1, monocyte chemotactic factor 1; OSM, oncostatin M; RANTES, regulated upon activation normal T cell expressed and presumably secreted; TNFα, tumour necrosis factor alpha; VEGF, vascular endothelial growth factor.

Principal component analysis on cytokine levels of synovial fluid, cartilage and *in vitro* produced tissue was performed to identify the existence of specific subsets of cytokines, indicating involvement in different biological processes. Clustering of specific cytokines was indeed very different between cartilage tissue and nonexpanded cells. In order to facilitate comparison of clusters between tissue and cells we chose to colour all cytokines belonging to the same cluster a certain colour (see Figure 4). Clustering in expanded cells was possibly even more distinct because here two-thirds of the variance could be explained by a large cluster of many cytokines (Figure 4).

### Synovial fluid

Principal component analysis of synovial fluid revealed three clusters of correlated cytokines (Table 4). Cluster 1 consisted of IL-1α, IL-7, IL-8, IL-10, TNFα and OSM, and explained 53% of variance with an eigenvalue of 5.9. Cluster 2 contained IL-1β, IL-4 and IL-13 (variance explained 27%, eigenvalue 2.9), and cluster 3 contained IL-6 and IFNγ (variance explained 10%, eigenvalue 1.1).

**Table 4:** **Principal component analysis of synovial fluid**

**Mediator**	**Cluster**		
	**1**	**2**	**3**
IL-7	0.997		
IL-10	0.966		
TNFα	0.938		
IL-8	0.874		
OSM	0.747		
IL-1α	0.661		
IL-1β		0.970	
IL-13		0.956	
IL-4		0.919	
IL-6			0.876
IFNγ			0.745
Eigenvalues	5.9	2.9	1.1
Variance explained (%)	53	27	10
Cronbach’s alpha	0.829	0.680	0.228

IFNγ, interferon gamma; IL, interleukin; OSM, oncostatin M; TNFα, tumour necrosis factor alpha.

### Cartilage tissue

A total of five clusters were identified that respectively explained 33%, 14%, 11%, 11% and 7% of variance (eigenvalues 6.2, 2.7, 2.1, 2.0 and 1.3 respectively; Table 5). Although some of the cytokines found in cluster 1 (IL-1α, IL-10, IL-13, TNFα, OSM, LIF, MCP1, RANTES) showed some overlap with cluster 1 identified in synovial fluid, mostly clusters were different. Components of cluster 2 were adiponectin, bFGF, HGF and VEGF; those of cluster 3 were IL-1β and IFNγ; those of cluster 4 were IL-6 and IL-8; and components of cluster 5 were IL-4, leptin and IL-1RA.

**Table 5:** **Principal component analysis of cartilage tissue**

**Mediator**	**Cluster**				
	**1**	**2**	**3**	**4**	**5**
IL-1α	0.947				
TNFα	0.926				
OSM	0.914				
RANTES	0.912				
IL-10	0.835				
IL-13	0.814				
MCP1	0.745				
LIF	0.693				
Adiponectin		0.846			
HGF		0.789			
bFGF		0.664			
VEGF		0.610			
IL-1β			0.963		
IFNγ			0.933		
IL-6				0.967	
IL-8				0.945	
IL-4					0.690
Leptin					0.508
IL-1RA					−0.466
Eigenvalues	6.2	2.7	2.1	2.0	1.3
Variance explained (%)	33	14	11	11	7
Cronbach’s alpha	0.772	0.024	0.196	0.272	0.001

bFGF, basic fibroblast growth factor; HGF, hepatocyte growth factor; IFNγ, interferon gamma; IL, interleukin; LIF, leukaemia inhibitory factor; MCP1, monocyte chemotactic factor 1; OSM, oncostatin M; RANTES, regulated upon activation normal T cell expressed and presumably secreted; TNFα, tumour necrosis factor alpha; VEGF, vascular endothelial growth factor.

### *In vitro* tissue formation

Principal component analysis of the cytokines produced by nonexpanded cells revealed five clusters that respectively explained 34%, 19%, 12%, 8% and 5% of variance (eigenvalues 7.1, 3.9, 2.5, 1.6 and 1.1 respectively; Table 6). Cluster 1 contained IL-1α, IL-1β, IL-10, OSM, MCP1 and IL-1RA; cluster 2 contained IL-4, IL-7, IL-13, IFNγ, leptin and HGF; cluster 3 contained IL-6, IL-8 and RANTES; cluster 4 contained adiponectin, bFGF and TNFα; and cluster 5 contained LIF, VEGF and IL-6Rα. Cytokines produced by expanded cells clustered in only four clusters (66%, 11%, 6% and 5% of variance explained with eigenvalues of 13.8, 2.4, 1.2 and 1.0 respectively; Table 7). Cluster 1 consisted of IL-1α, IL-1β, IL-1RA, IL-4, IL-7, IL-10, IL-13, TNFα, IFNγ, OSM, adiponectin, leptin, bFGF and HGF, and explained most of the variance; cluster 2 of IL-6, IL-8, LIF, MCP1 and RANTES; cluster 3 of VEGF; and cluster 4 consisted of IL-6Rα. None of the cytokines or clusters was related to GAG/DNA production.

**Table 6:** **Principal component analysis of nonexpanded chondrocytes**

**Mediator**	**Cluster**				
	**1**	**2**	**3**	**4**	**5**
OSM	0.954				
IL-10	0.932				
IL-1β	0.889				
MCP1	0.859				
IL-1α	0.804				
IL-1RA	0.700				
Leptin		0.880			
HGF		0.857			
IL-13		0.817			
IL-4		0.772			
IL-7		0.754			
IFNγ		0.661			
IL-8			−0.856		
IL-6			0.808		
RANTES			0.682		
bFGF				0.764	
Adiponectin				0.750	
TNFα				0.667	
VEGF					0.741
LIF					0.619
IL-6R					0.588
Eigenvalues	7.1	3.9	2.5	1.6	1.1
Variance explained (%)	34	19	12	8	5
Cronbach’s alpha	0.152	0.392	−3.235	0.032	0.068

bFGF, basic fibroblast growth factor; HGF, hepatocyte growth factor; IFNγ, interferon gamma; IL, interleukin; LIF, leukaemia inhibitory factor; MCP1, monocyte chemotactic factor 1; OSM, oncostatin M; RANTES, regulated upon activation normal T cell expressed and presumably secreted; TNFα, tumour necrosis factor alpha; VEGF, vascular endothelial growth factor.

**Table 7:** **Principal component analysis of expanded chondrocytes**

**Mediator**	**Cluster**			
	**1**	**2**	**3**	**4**
IL-4	0.980			
IL-1β	0.976			
Adiponectin	0.971			
Leptin	0.963			
TNFα	0.958			
IL-1α	0.954			
IL-7	0.954			
IL-10	0.953			
HGF	0.944			
IFNγ	0.942			
IL-13	0.940			
IL-1RA	0.926			
OSM	0.912			
bFGF	0.845			
IL-8		0.817		
IL-6		0.788		
RANTES		0.671		
LIF		0.635		
MCP1		0.541		
VEGF			0.847	
IL-6R				0.914
Eigenvalues	13.8	2.4	1.2	1.0
Variance explained (%)	66	11	6	5
Cronbach’s alpha	0.812	0.293	n.a.	n.a.

bFGF, basic fibroblast growth factor; HGF, hepatocyte growth factor; IFNγ, interferon gamma; IL, interleukin; LIF, leukaemia inhibitory factor; MCP1, monocyte chemotactic factor 1; n.a., not available; OSM, oncostatin M; RANTES, regulated upon activation normal T cell expressed and presumably secreted; TNFα, tumour necrosis factor alpha; VEGF, vascular endothelial growth factor.

## Discussion

To the best of our knowledge, this is the first report directly comparing cytokine presence in synovial fluid and in cartilage and cytokine production by isolated chondrocytes from donors without joint pathology, patients with symptomatic cartilage defects and patients with OA. Several proinflammatory, proangiogenic and pro-repair cytokines were elevated in donors with symptomatic cartilage defects and OA. Especially in diseased joints, local concentrations of inflammatory cytokines present in the cartilage tissue were markedly higher than in synovial fluid. Furthermore, cytokine levels were greatly increased during *in vitro* tissue production by isolated chondrocytes. Interestingly, different cytokines were regulated by the underlying pathology status in synovial fluid, cartilage tissue and isolated chondrocytes. This was further supported by clustering of correlated cytokines, which was different between synovial fluid, cartilage tissue and cultured cells, indicating that the cytokines were involved in different biological processes depending on the biological sample analysed.

We observed high concentrations of inflammatory (IFNγ, OSM, IL-6) and pro-repair (IL-13) cytokines in synovial fluid of joints with cartilage defects and OA. The increased presence of these cytokines may be one of the reasons for the inferior clinical results observed for autologous chondrocyte implantation in patients with longer existing degenerative defects and OA [18,19]. Elevated levels of inflammatory mediators have previously been demonstrated in the synovial fluid of patients with joint trauma and OA [20], and synovial fluids from these patients have been shown to hamper cartilage regeneration [6,21]. However, the composition of the synovial fluid in patients with symptomatic cartilage defects had hardly been characterised until now. In joint trauma, levels of inflammatory cytokines have been shown to be very high initially and decrease over the course of weeks [22]. Likewise, we found higher levels of IL-13 and IFNγ in the synovial fluids of cartilage defect donors than in OA donors. A recent study by Heard and colleagues [23] also evaluated the presence of cytokines in synovial fluid of patients with severe and mild OA. Mild OA was defined as cartilage damage <2 Outerbridge score on arthroscopy (which means the cartilage damage was partial thickness at most), which is milder than the patients with cartilage defects included in this study that were mostly full thickness cartilage defects. They reported on a similar increase in IL-6 with increasing cartilage damage. However, contrary to our results they did not find higher levels of IFNγ and IL-13, possibly because cartilage damage in their mild OA groups was less severe than in our cartilage defects group. Interestingly, they were also able to use principal component analysis to identify two main components by which they could make a distinction between healthy and OA patients. Unfortunately, due to the limited sample size we were not able to do the same.

Generally, inflammatory mediators are thought to negatively affect cartilage integrity, but this may be too simplistic a view. OSM present in the synovial fluid is indeed known to inhibit *in vitro* cartilage regeneration [21]. However, IFNγ can also inhibit IL-1-induced matrix metalloproteinase-13 expression in healthy and osteoarthritic chondrocytes [24,25], and we have recently shown that IL-6 can also stimulate cartilage matrix formation under specific conditions [15]. Effects of cytokines may therefore not only depend on the presence of other cytokines with opposite effects, but also on the disease status, and effects on chondrocytes may be different than on other cell types. The elevated presence of IL-13 in cartilage defects had not been reported until now. IL-13 is a T-helper 2 cytokine that mediates alternative macrophage activation, a process that is now recognised to play a role not only in immunity, but also in tissue homeostasis and repair [26]. IL-13 is classically T-helper 2 derived, but it can also be produced by various other cells and mediates fibrotic aspects of innate immune activation through both transforming growth factor beta-dependent and transforming growth factor beta-independent pathways in pulmonary, hepatic, renal, dermal and gastrointestinal inflammation and fibrosis [27]. The direct effects of IL-13 on chondrocytes are not well known, but it has been shown to prevent collagen release induced by IL-1α and OSM in bovine nasal cartilage [28]. The effects on inflammation and cartilage integrity have been evaluated in various murine arthritis models, but not in OA models or defect models. Decreased cartilage destruction and chondrocyte apoptosis were found with local overexpression of IL-13, but both decreased inflammation [29] and increased inflammation [30] were reported depending on the model employed. Furthermore, microarray analysis of peripheral blood cells of patients with early OA (limited damage to the cartilage surface at arthroscopy, traumatic chondral defects excluded) identified IL-13 receptor as one of the six genes significantly downregulated [31]. It may be interesting to explore whether IL-13 receptor downregulation also occurs in patients with focal cartilage defects and whether this is predictive for outcome.

We also observed that the concentrations of the inflammatory cytokines IL-1α, IL-1β, IL-6, TNFα, IFNγ and OSM in diseased cartilage tissue were distinctly elevated compared with those found in synovial fluid. This may indicate that their role in cartilage turnover in these conditions may be more substantial than can be deduced from only evaluating synovial fluid concentrations. All of these cytokines can have catabolic effects on cartilage and chondrocytes [32]. This also indicates that although the synovial membrane has been commonly implied in governing the inflammatory process leading to cartilage destruction, it appears that other tissues, including the cartilage itself, may guide this. The origin of these cytokines, however, is unclear. While IFNγ and IL-1β indeed were found to be actively produced by OA cartilage in *in vitro* explant cultures [8], IL-6 and OSM have been found to be produced by OA synovial lining but not by cartilage. Other tissues in the joint, such as the underlying bone, may therefore also have produced some of the factors we measured in native cartilage tissue.

In cartilage tissue an increased presence of VEGF was found in cartilage debrided from defects and to a lesser extent also in OA cartilage compared with healthy cartilage. We did not evaluate the presence of VEGF in synovial fluid, but in a recent study in end-stage OA patients undergoing knee arthroplasty [20], in which the levels of IL-6, TNFα and VEGF were measured, almost identical levels of IL-6 and TNFα as in the current study and also high levels of VEGF were present. Additional to its stimulatory effects on angiogenesis, VEGF may directly influence cartilage. Increased presence of VEGF and its receptors have been previously found in OA cartilage tissue [33]. VEGF has been implicated in OA pathogenesis since injection of VEGF into mice knees induced OA [34] and VEGF immunolocalisation correlated with cartilage destruction in several experimental animal models [35]. More recently it has not only been detected in late OA but also in early stages of OA [36]. VEGF has also been implicated to play a role in regeneration because it was shown to inhibit aggrecan and collagen II synthesis [37] and inhibition of VEGF using a monoclonal antibody improved cartilage regeneration [38]. Our results also support a role for VEGF in cartilage defects, although small quantities were also found in healthy tissue.

Expansion is known to lead to a loss of chondrocyte phenotype [39]. Therefore it may not be surprising that also the production of cytokines changes with expansion. However, culturing chondrocytes generally led to a massive induction of cytokines released, which has not been shown before. The role of this generalised upregulation of cytokine production in cell culture is not clear, nor what mechanism is involved. In cartilage, like in general wound healing, a temporary inflammatory activation has been suggested to be crucial for chondrogenic differentiation [40]. However, despite this general upregulation, differences between healthy and diseased chondrocytes remained. Nonexpanded chondrocytes obtained from debrided defect cartilage produced more IL-1β than healthy and OA chondrocytes, and IL-6, IL-8 and LIF were differentially produced by expanded chondrocytes. IL-6 and IL-8 are two key cytokines known to be secreted by senescent cells, something known as the senescence-associated secretory phenotype [41,42]. Both replication as well as stresses such as oxidative stress, DNA damage reagents and several cytokines, including IL-6 and IL-8 [41,42], can induce DNA damage and telomere shortening. A stress-induced rather than replication-induced senescence-like phenotype has been reported in OA chondrocytes, especially near osteoarthritic lesions [43,44]. Expansion of the OA chondrocytes may have amplified the already present DNA damage, thereby increasing the senescence-associated secretion of cytokines. Differences in age between donors with cartilage defects and those with OA in particular may also have contributed to differences in cytokine levels [45].

Chondrocyte cell cultures are frequently used to study inflammatory mechanisms involved in cartilage metabolism and OA [46,47]. However, cytokine profiling as well as clustering of correlated cytokines were distinctly different between native tissue and cartilage cells in culture, even when the cells were not expanded in culture and had a differentiated chondrogenic phenotype. Correlated cytokines identified by principal component analysis are thought to indicate an underlying biological process. The large difference in clustering between cartilage tissue and chondrocyte culture indicates that results obtained from studying the effects of cytokines on cartilage explants probably cannot be extrapolated to tissue regeneration cultures. Likewise, cytokines reported to have negative effects on cartilage integrity in OA may not necessarily also be detrimental to cartilage repair in autologous chondrocyte implantation. The different clustering of cytokines released by chondrocytes in culture compared with in native tissue may further indicate a general response to culture. In this regard, the loss of diversity with the expansion of chondrocytes (the first cluster explained more than three-quarters of the variance in expanded cells) further calls for caution with extrapolation of the results of chondrocyte culture to clinical OA, even if the cells are redifferentiated as in our culture model.

Despite the increased release of inflammatory cytokines by chondrocytes obtained from cartilage debrided from defects and OA cartilage, *in vitro* cartilaginous tissue formation with these chondrocytes was at least as good as or even superior to regeneration with healthy chondrocytes in terms of GAG production, confirming other studies [48-51]. In fact, GAG production is increased in mild to moderate severity OA cartilage [52,53].

Regression analysis did not reveal any cytokines or clusters of cytokines that correlated with cartilage matrix production. Cytokines could have dual or opposing effects. Perhaps other, here unmeasured, mediators are important for regeneration, or cytokine release during cell culture reflects a more general response to culture rather than a pathophysiological process. Unfortunately we did not measure all of these cytokines produced during regeneration in synovial fluid as we started out by measuring cytokines in synovial fluid that were previously found in pathological joints and were suggested to play a role in cartilage degeneration. Not much was known on chondrocyte-based production, so we measured a much broader panel of cytokines here. We agree that in retrospect it would have been interesting to see whether cytokines differentially regulated in chondrocytes could be traced back to synovial fluid, but unfortunately the amount of synovial fluid specimens especially from healthy joints is very limited and therefore we cannot perform more measurements.

Further research is needed to clarify the role, if any, of cytokines during regeneration.

Limitations of our study include the fact that donors were not age or gender matched. We therefore cannot exclude that at least part of the observed higher concentration of inflammatory markers was age associated. However, patients with symptomatic cartilage defects are young and predominantly male [54] whilst OA generally affects older patients, so these differences between the populations are inherent to the affliction. Secondly, we mostly saw higher inflammation in patients with cartilage defects; therefore, if anything, eliminating age-associated inflammation will probably result in even larger differences. Further, the collection of tissue from donors without joint pathology was performed post mortem as opposed to the intraoperative collection of OA and cartilage defect synovial fluid. Synovial fluid collection was performed by needle aspiration and not lavage, thereby eliminating the need for correction to protein and as soon as possible, usually several hours and up to a maximum of 24 hours post mortem. Furthermore, the donors were kept cooled until harvest, but we cannot exclude that some changes in composition might have occurred. However, cartilage is not vascularised and remains very much unaltered during the first 24 hours [55] and previous work from our group showed no difference in viability between freshly isolated chondrocytes from healthy, postmortem, and grade III cartilage defect tissue [10].

## Conclusions

Taken together, we showed that several proinflammatory, proangiogenic and pro-repair cytokines were elevated in patients with symptomatic cartilage defects and OA, and that different cytokines were upregulated in synovial fluid, cartilage tissue and isolated cartilage cells. Research into the mechanisms governing this differential release may shed light on the actual role of these factors in degeneration and regeneration of cartilage tissue.

## Abbreviations

bFGF: basic fibroblast growth factor
GAG: glycosaminoglycan
HGF: hepatocyte growth factor
IFNγ: interferon gamma
IL: interleukin
LIF: leukaemia inhibitory factor
MCP1: monocyte chemotactic factor 1
OA: osteoarthritis
OSM: oncostatin M
PBS: phosphate-buffered saline
RANTES: regulated upon activation normal T cell expressed and presumably secreted
TNFα: tumour necrosis factor alpha
VEGF: vascular endothelial growth factor
